# Beamspace Spatial Smoothing MUSIC DOA Estimation Method Using Dynamic Metasurface Antenna

**DOI:** 10.3390/e27040335

**Published:** 2025-03-24

**Authors:** Lilong Hou, Liang Jin, Kaizhi Huang, Shuaifang Xiao, Yangming Lou, Yajun Chen

**Affiliations:** Wireless Communication Technology Office, Information Engineering University, Zhengzhou 450002, China; lilong.hou@outlook.com (L.H.); huangkaizhi@tsinghua.org.cn (K.H.); xiaoshuaifang.2007@tsinghua.org.cn (S.X.); louyangming1991@outlook.com (Y.L.); chenyajun_cool@126.com (Y.C.)

**Keywords:** two-dimensional direction-of-arrival estimation, 2D DOA, beamspace space smoothing music, dynamic metasurface antenna, DMA

## Abstract

The Direction-of-Arrival (DOA) estimation method using traditional array antennas cannot dynamically adjust the observation angle range based on the Region of Interest (ROI), which leads to limited estimation accuracy and high computational complexity. To address the above issue, this paper proposes a Beamspace Spatial Smoothing MUltiple SIgnal Classification (BSS-MUSIC) DOA estimation method using a Dynamic Metasurface Antenna (DMA). Specifically, we propose a new DMA model with a single RF chain and exploit its flexibility to design a time-division data reception scheme. Based on this scheme, we dynamically select the ROI and increase the beam density in the ROI with an appropriate number of beam patterns. Next, a BSS algorithm is proposed to decohere the multipath signals in beamspace without reverting to the element space. Subsequently, we convert the 2D DOA estimation into two 1D beamspace MUSIC DOA estimations. After pairing the elevation and azimuth angles, the complex gains of each path are derived. Simulation results show that the proposed method can achieve higher estimation accuracy with lower computational complexity.

## 1. Introduction

Antenna array signal processing has extensive applications in radar, sonar, navigation, and wireless communications. As a key technique, Direction of Arrival (DOA) estimation has become an important research topic in this field [1,2]. However, the performance of DOA estimation is inevitably compromised by the presence of multipath effects, causing destructive interference that represents a significant challenge to the effectiveness of DOA estimation algorithms.

Many decoherence methods for effectively mitigating the deleterious effects of coherent multipath signals have been reported in the literature. These can be divided into two categories. First, algorithms that are insensitive to whether or not the signal is coherent include Maximum Likelihood (ML) [3], Weighted Subspace Fitting (WSF) [4], and Compressed Sensing (CS) [5]. These algorithms can be directly applied to estimate the DOA of coherent multipath signals. For example, Wu et al. proposed a method that combines ML and Orthogonal Matching Pursuit (OMP) for DOA estimation [3]. Wang et al. proposed a robust weighted block sparse reconstruction DOA estimation method using the optimal WSF [4]. Unfortunately, ML and WSF both have high computational complexity compared to Iterative Quadratic Maximum Likelihood (IQML) and Method of Direction Estimation (MODE). Li et al. conducted a comparative study between the IQML and MODE algorithms for DOA estimation of narrowband impinging signals, finding that MODE demonstrated superior performance [6]. The second class of algorithms is sensitive to coherence, and includes examples such as Multiple Signal Classification (MUSIC) [7], Root-MUSIC [8], and Estimation of Signal Parameters via Rotational Invariance Techniques (ESPRIT) [9]. These algorithms perform subspace classification, but cannot be used directly, requiring the Spatial Smoothing (SS) preprocessing algorithm. You et al. used the MUSIC algorithm based on Forward Spatial Smoothing (FSS) to estimate the coherent DOA of signals [10]. Li et al. used Forward/Backward Spatial Smoothing (FBSS) with Root-MUSIC to estimate the coherent DOA of signals using a moving co-prime array [11]. Pan et al. proposed an ESPRIT variant using a simplified spatial smoothing technique to estimate DOA in coherent scenarios [12]. Ning et al. used the ESPRIT algorithm with double parallel linear arrays to estimate 1D DOA [13].

While 1D DOA estimation is commonly used for simple localization tasks, 2D DOA estimation offers more comprehensive information by incorporating both azimuth and elevation angles, making it particularly useful for applications requiring accurate source localization in three-dimensional space. MUSIC, ESPRIT, ML, and WSF can be extended to 2D DOA estimation; however, both ML and WSF suffer from high computational complexity in 2D estimation, making them impractical for real-world applications due to the optimization of multimodal functions and high associated computational costs [14]. For these reasons, ESPRIT and MUSIC are commonly used for 2D DOA estimation. Qiu et al. proposed an innovative structure for a sparse co-prime MIMO radar and developed a dual-resolution 2D unitary ESPRIT method for DOA estimation [15]. Wang and Grishin proposed the 2D SS-MUSIC method for estimating the elevation and azimuth using URA. In this approach, an M×N array is divided into overlapping rectangular subarrays of size $M_{sub}\times N_{sub}$ ($M_{sub}<M$, $N_{sub}<N$) [16,17].

However, the process of finding the 2D search peak is computationally expensive; this motivates the exploration of more efficient techniques that can reduce the overall computational burden, such as converting the 2D spectral peak search to 1D search. Wang et al. proposed a tree-structured algorithm based on the 1D MUSIC algorithm for estimating the 2D DOAs of coherent signals incident on Uniform Rectangular Arrays (URA) [18]. Ertan et al. transformed the 2D L-shaped array into a 1D array, followed by selecting the appropriate subarray to perform 1D SS-MUSIC estimation [19]. Zhang et al. proposed a novel low-complexity 2D DOA estimation approach based on 1D MUSIC using the unfolded co-prime L-shaped array [20].

Despite these advancements, the above-mentioned DOA estimation methods using traditional array antennas suffer from limited accuracy and high computational complexity due to their fixed structures and low Degrees of Freedom (DOF). This results in a fixed observation angle range that cannot be dynamically adjusted based on the Region of Interest (ROI). To address these issues, recent research has focused on beamspace signal processing techniques based on more adapted array, which allow for dynamic adjustment of observation angles. Dong et al. used a Lens Antenna Array (LAA) to determine the observation angles based on the incident beams, and then applied the beamspace MUSIC algorithm to perform DOA estimation in a lower-dimensional space [21]. Guo et al. proposed a hybrid antenna array consisting of analog subarrays to provide a flexible beam-scanning scheme for DOA estimation [22,23]. Chen et al. proposed a novel DOA estimation paradigm based on the convolutional beamspace approach using a hybrid antenna array, which effectively reduces the number of required RF chains while enhancing the precision of beamspace ESPRIT estimation [24,25]. Nevertheless, these array antennas still require many high-performance RF chains and phase shifters, causing significant hardware complexity that limits their practical applications.

Metamaterial antennas have decreased the dependency on RF chains and phase shifters. Additionally, metamaterial array elements can precisely control the parameters of electromagnetic waves, thereby improving the DOF [26]. In Non-Line-of-Sight (NLOS) scenarios, the metamaterial technique can be employed to enhance communication performance. Atomic norm-based methods are applied to estimate the DOA using reconfigurable intelligent surfaces (RIS) [27,28]. Wang et al. proposed an RIS-assisted cascade channel estimation method, which is cast as sparse signal recovery problem and uses the CS algorithm to estimate the DOA [29]. Chen et al. proposed an RIS-aided multi-user mmWave MIMO system and formulated the channel estimation problem as a sparse channel matrix recovery problem, which enables DOA estimation with limited training overhead [30]. Wang et al. proposed an innovative approach to create temporal-domain multidimensional received signals for estimating the DOA of the paths from the users to the RIS [31]. Nevertheless, CS algorithms such as the OMP algorithm are typically used in single-snapshot scenarios; additionally, RIS reflects the impinging signal in the desired direction to help improve the transmission performance between user and transceiver. On the other hand, metamaterial-based antennas, such as dynamic metasurface antenna (DMA), could be deployed at the transceiver to enhance the signal reception quality.

A DMA consists of tunable metamaterial antenna elements packed into compact microstrips, ensuring that the signal flows within the microstrip [32,33]. Meanwhile, DMA provides beam tailoring capabilities and facilitates the processing of signals in the analog domain in a dynamically configurable manner. Therefore, DMA has vital applications in various domains, including reconfigurable beamforming [34], physical-layer key generation [35], application to synthetic aperture radar systems [36], and ultra-massive MIMO communications for 6G [32]. DMA significantly enhances signal reception efficiency and shows considerable potential in DOA estimation [37]. Lou et al. exploited DMA to construct an observation matrix to improve signal reception performance, then used the minimum atomic norm algorithm to estimate the DOA [38]. However, the computational complexity of this approach is relatively high. Wang et al. and Chen et al. respectively exploited DMA to construct beam patterns and observe signals using different weighting algorithms, and then applied the beamspace MUSIC algorithm to obtain the DOA [39,40]. However, the beam patterns are not designed in relation to the incident signal, which limits the performance improvement.

The MUSIC algorithm can be easily applied to DMA, and can achieve superior DOA estimation performance due to its low computational complexity and excellent resolution [41]. Therefore, in this paper we propose the Beamspace Spatial Smoothing MUltiple SIgnal Classification (BSS-MUSIC) DOA estimation method using DMA. Our main contributions are listed as follows:

- We propose a DMA-assisted receiver system model with a single RF chain. Based on this model, we establish a time-division data reception scheme to sequentially capture the received signal data. The observation angle range can be dynamically adjusted using this scheme. Within the adjusted range, the pattern can be rapidly changed during a single pilot symbol period, enabling the reception of signals equivalent to those obtained by a multi-RF chain system.
- To reduce computational complexity and improve the accuracy of the DOA estimation algorithm, we dynamically select the ROI and increase the beam density in the ROI with an appropriate number of beam patterns based on the time-division data reception scheme. Moreover, the BSS-MUSIC algorithm converts 2D DOA estimation into two 1D beamspace MUSIC DOA estimations, which helps to reduce the angular search space. Next, beamspace spatial smoothing effectively utilizes the array aperture to improve the estimation accuracy of the algorithm, achieving multipath signals decorrelation. Finally, the beamspace MUSIC algorithm accurately estimates the DOAs.
- The feasibility of the proposed method is verified through several simulated experiments. The simulation results illustrate that our method offers improved estimation accuracy and reduced computational complexity. Furthermore, it effectively minimizes the required number of RF chains while improving the DOF.

The remainder of this paper is organized as follows: Section 2 presents the DMA-assisted receiving system model; Section 3 presents the BSS-MUSIC DOA estimation framework; Section 4 exhibits numerical results and discusses the effectiveness of the proposed method; finally, conclusions are provided in Section 5.

For notation, we use ${\left(\cdot \right)}^{T}$ to imply transpose and ${\left(\cdot \right)}^{H}$ to denote the conjugate transpose; in addition, ${\left(\cdot \right)}^{†}$ denotes the Moore–Penrose pseudoinverse, ${∥\cdot ∥}_{2}$ is the $l_{2}$ norm, and C denotes the complex field.

## 2. DMA-Assisted Receiving System Model

**Figure 1:**
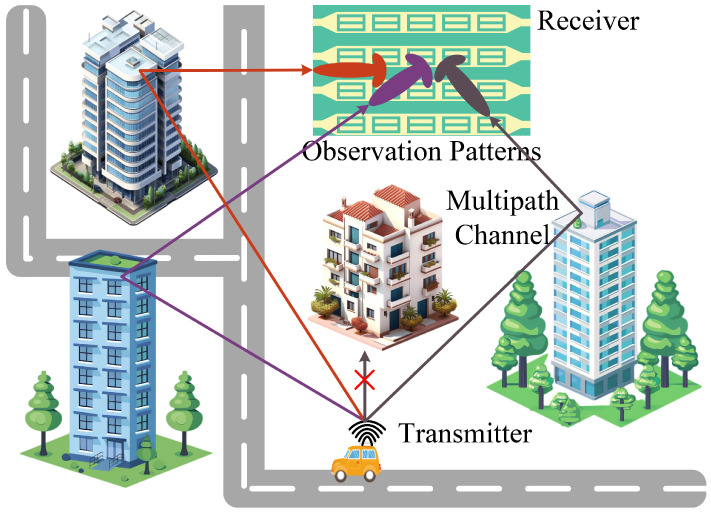
Multipath propagation model. The pilot signal from the user reaches the DMA through multiple paths, and the DMA constructs multiple observation patterns to observe the signal.

In this paper, we propose a DMA-assisted receiving system for DOA estimation, where the DMA is deployed at the base station. As shown in Figure 1, the transmitted pilot signal from a single-antenna user passes through a multipath environment before reaching the DMA receiver, with the assumption that Line-of-Sight (LOS) propagation is neglected. The DMA uses a series of weighted vectors to form the array patterns for observing the multipath channel.

**Figure 2:**
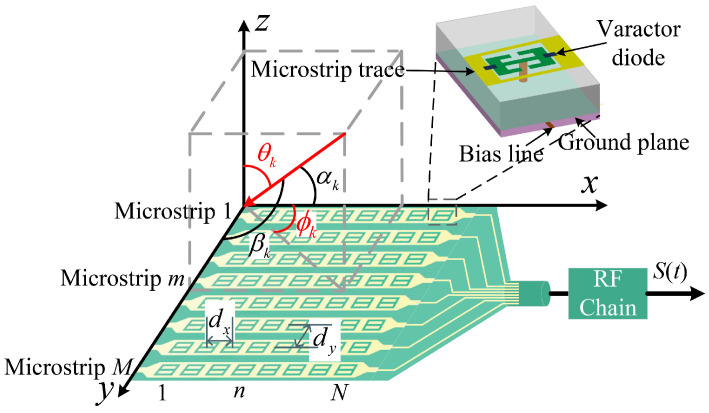
DMA antenna schematic diagram. All *M* microstrips are connected to an RF chain; $\theta_{k}$ and $\phi_{k}$ respectively represent the elevation and azimuth of the *k*th multipath signal, while $\alpha_{k}$ and $\beta_{k}$ are the angles with the *x*-axis and *y*-axis, respectively.

As shown in Figure 2, the DMA is assumed to be an M×N uniform rectangular array, and we consider ideal continuous phase shifts. The DMA consists of *M* microstrips, with each microstrip containing *N* radiating elements made of reconfigurable metamaterial. The DMA is located along the *x*-axis and *y*-axis with interval distances $d_{x}$ and $d_{y}$, respectively. The microstrips or elements are separated by a half-wavelength, i.e., $d_{x}=d_{y}=\lambda /2$. The array elements individually manipulate the phase shifts of the impinging signals in a preferred way, then the phase-shifted signals flow into a single RF chain in combination mode.

Assume that K(K<min(M,N)) far-field narrowband multipath signal impinging on the DMA, and let $\alpha_{k}$ and $\beta_{k}$ be the angles between the *k*th multipath signal arrival direction and the *x*-axis or *y*-axis, respectively, where k=1,2,⋯,K. $\theta_{k}$ and $\phi_{k}$ represent the elevation and azimuth of the *k*th multipath signal, $\theta_{k}\in \left[0,90^{\circ }\right]$, $\phi_{k}\in \left[-180^{\circ },180^{\circ }\right)$. Assume that the signals arrive in different 2D DoAs as $\left(\theta_{1},\phi_{1}\right),\left(\theta_{2},\phi_{2}\right),\cdots ,\left(\theta_{K},\phi_{K}\right)$.

All channels are assumed to be block-faded and to remain unchanged within each frame, with perfect estimation at the receiver through pilot symbols. We utilize the widely used Saleh–Valenzuela multipath channel model [42]. Specifically, the channel model between the DMA and the user along the *x*-axis or *y*-axis can be expressed by

(1) $$H_{x}=\sum_{k=1}^{K}h_{k}a_{x}\left(\theta_{k},\phi_{k}\right),$$

(2) $$H_{y}=\sum_{k=1}^{K}h_{k}a_{y}\left(\theta_{k},\phi_{k}\right),$$

where the $h_{k}$ represents the normalized complex gains for the corresponding multipaths. The normalized array steering vectors $a_{x}\left(\theta_{k},\phi_{k}\right)$ and $a_{y}\left(\theta_{k},\phi_{k}\right)$, also known as the manifold vectors, represent the *x*-axis and *y*-axis steering vectors and are provided by

(3) $$a_{x}\left(\theta_{k},\phi_{k}\right)={\left[1,e^{j2\pi d_{x}\cos\alpha_{k}/\lambda },\cdots ,e^{j2\pi d_{x}\cos\alpha_{k}\left(N-1\right)/\lambda }\right]}^{T},$$

(4) $$a_{y}\left(\theta_{k},\phi_{k}\right)={\left[1,e^{j2\pi d_{y}\cos\beta_{k}/\lambda },\cdots ,e^{j2\pi d_{y}\cos\beta_{k}\left(M-1\right)/\lambda }\right]}^{T},$$

where $\cos\alpha_{k}=\sin\theta_{k}\cos\phi_{k}$ and $\cos\beta_{k}=\sin\theta_{k}\sin\phi_{k}$ are derived from the geometric relationship, as shown in Figure 2. Then, we can obtain the manifold matrix of the *x*-axis and *y*-axis as $A_{x}\left(\theta ,\phi \right)=\left[a_{x}\left(\theta_{1},\phi_{1}\right),\cdots ,a_{x}\left(\theta_{K},\phi_{K}\right)\right]\in C^{N\times K}$, $A_{y}\left(\theta ,\phi \right)=\left[a_{y}\left(\theta_{1},\phi_{1}\right),\cdots ,a_{y}\left(\theta_{K},\phi_{K}\right)\right]\in C^{M\times K}$. For ease of observation, we abbreviate these symbols as $A_{x}$ and $A_{y}$.

Assume that the far-field narrowband BPSK modulated pilot signal is $s\left(t\right)=\exp\left(j2\pi f_{c}t+\psi \right)$, 0<t<T, ψ=0 or ψ=π, where *T* is a pilot symbol duration. Then, the pilot sequence can be represented as $s=\left[s_{1},\cdots ,s_{l},\cdots ,s_{L}\right]\in C^{1\times L}$. When the *l*th symbol is sent, the (m,n) element data can be expressed as

(5) $$x_{m,n}\left(l\right)=\sum_{k=1}^{K}e^{j2\pi d_{x}\cos\alpha_{k}\left(n-1\right)/\lambda }e^{j2\pi d_{y}\cos\beta_{k}\left(m-1\right)/\lambda }h_{k}s_{l}+W_{m,n}\left(l\right).$$

Hence, the element-space signals of the *m*th row and *n*th column with respect to the *x*-axis and the *y*-axis can be expressed as

(6) $$X_{m}^{e}=A_{m}^{x}hs+W_{m},$$

(7) $$Y_{n}^{e}=A_{n}^{y}hs+W_{n},$$

where $h={\left[h_{1},h_{2},\cdots ,h_{k}\right]}^{T}$, $A_{m}^{x}=A_{x}\times \mathrm{diag}\left[A_{y}\left(m,:\right)\right]=A_{x}\Phi_{y}^{m-1}$, $A_{n}^{y}=A_{y}\times \mathrm{diag}\left[A_{x}\left(n,:\right)\right]=A_{y}\Phi_{x}^{n-1}$, and $\Phi_{y}^{m-1}=\mathrm{diag}\left(e^{j2\pi d_{y}\cos\beta_{1}\left(m-1\right)/\lambda },\cdots ,e^{j2\pi d_{y}\cos\beta_{K}\left(m-1\right)/\lambda }\right)\in C^{K\times K}$, $\Phi_{x}^{n-1}=\mathrm{diag}\left(e^{j2\pi d_{x}\cos\alpha_{1}\left(n-1\right)/\lambda },\cdots ,e^{j2\pi d_{x}\cos\alpha_{K}\left(n-1\right)/\lambda }\right)\in C^{K\times K}$. Assuming that the noise follows a circularly symmetric complex Gaussian distribution with zero mean, there is statistical independence between the signals and noise.

Moreover, due to multipath signal coherence, a spatial smoothing algorithm is required to decorrelate the signals. Therefore, an additional degree of smoothing is provided by using subrows or subcolumns [43]. We divide $X_{m}$ into two overlapping subrows, which can be defined as follows:

(8) $$X_{m}^{e}=\left[\begin{array}{c} X_{m1}^{e}\\ X_{m}^{e}\left(N,:\right) \end{array}\right]=\left[\begin{array}{c} X_{m}^{e}\left(1,:\right)\\ X_{m2}^{e} \end{array}\right],$$

where $X_{m1}^{e}$ and $X_{m2}^{e}$ are the subrow data of the *m*th row, provided by

(9) $$X_{mi}^{e}=A_{11}^{x}\Phi_{x}^{i-1}\Phi_{y}^{m-1}hs+W_{mi},$$

where $A_{11}^{x}=A_{x}\left(1:N-1,:\right)$ is the manifold matrix of the first subrow in the first row.

The metamaterial elements of the DMA rapidly change their weight in each signal symbol by the weighting matrix $\Omega_{x}$, then form differentiate observation patterns to observe the multipath signals. After collecting all observation data, the output of the subrow can be expressed as

(10) $$X_{m1\setminus m2}^{B}=\Omega_{x}^{H}X_{m1\setminus m2}^{e}.$$

Similarly, the subcolumn data of the *n*th column can be expressed as

(11) $$Y_{nj}^{e}=A_{11}^{y}\Phi_{y}^{j-1}\Phi_{x}^{n-1}hs+W_{nj},$$

where $A_{11}^{y}=A_{y}\left(1:M-1,:\right)$ is the manifold matrix of the first subcolumn in the first column. Then, the output of the subcolumn can be expressed as

(12) $$Y_{n1\setminus n2}^{B}=\Omega_{y}^{H}Y_{n1\setminus n2}^{e}.$$

However, these beamspace data cannot be used to estimate the DOA with the existing element-space MUSIC algorithm. Additionally, because the DMA in this paper has only a single RF chain, the beamspace MUSIC algorithm, which is commonly used with traditional array antennas, cannot be applied directly. Therefore, the next section describes how to estimate the DOA from the beamspace data, as shown in Equations (10) and (12).

## 3. BSS-MUSIC DOA Estimation Framework Based on DMA

The BSS-MUSIC DOA estimation algorithm is demonstrated in this section. First, we design a time-division data reception scheme to observe the multipath signals. Then, the beamspace spatial smoothing algorithm is proposed to decohere the multipath signals. Finally, the beamspace MUSIC algorithm is employed to estimate the DOAs and complex gain.

### 3.1. Design of Time-Division Data Reception Scheme Based on the DFT Algorithm

As the proposed DMA model has only a single RF chain, the beamspace MUSIC algorithm cannot be used directly. Therefore, it is essential to design a time-division data reception scheme in order to flexibly capture the necessary beamspace data. This scheme is based on the DFT algorithm, which has excellent beamforming capability.

#### 3.1.1. Implementation of Time-Division Data Reception Scheme Using DMA

The process of finding the 2D peak by using 2D subarrays in 2D DOA estimation has high computational complexity; therefore, we convert the 2D estimation to two 1D estimations to reduce the complexity. The time-division data reception scheme divides the DMA into 1D subarrays. Each row is divided into two overlapping subarrays, which helps to enhance the effective aperture. Subarrays from different rows can work together to complete spatial smoothing and sequentially utilize the RF chain by leveraging the fast on/off switching behavior of the DMA.

**Figure 3:**
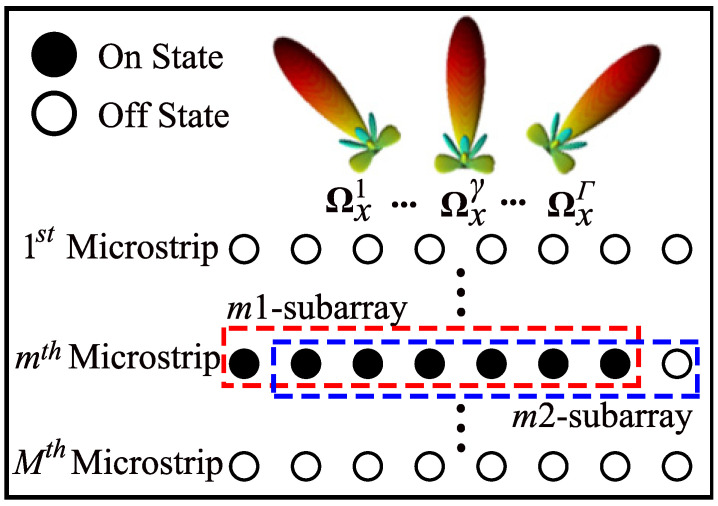
One-dimensional subarray partitioning scheme. The the on/off nature of the DMA is utilized to form multiple subarrays, each of which sequentially perceives the signals. When in the ‘on’ state, the subarray elements use the weighting matrix $\Omega_{x}$ to form diverse radiation patterns.

Each metamaterial element acts as a resonant electrical circuit, with its frequency response described by a Lorentzian equation. To implement the transition between the ‘on’ and ‘off’ states of DMA elements, the resonance frequency is toggled between the operating frequency and a frequency significantly different from it [33,44]. The selected elements in the ‘on’ state operate at the operating frequency, rapidly modifying the phase of the incident signal, while the selected elements in the ‘off’ state operate at a significantly different frequency from the operating frequency, resulting in no output from these elements [45]. Additionally, by exploiting the fast switching speed property of DMAs, which is faster compared to a single symbol period [46], we can assume that one symbol period is sufficient for all subarrays to adjust their elements and alter the radiation pattern 2×M×Γ times. As illustrated in Figure 3, the m1—subarray elements are in the ‘on’ state, forming diverse radiation patterns that allow for observation of the impinging signals.

**Figure 4:**
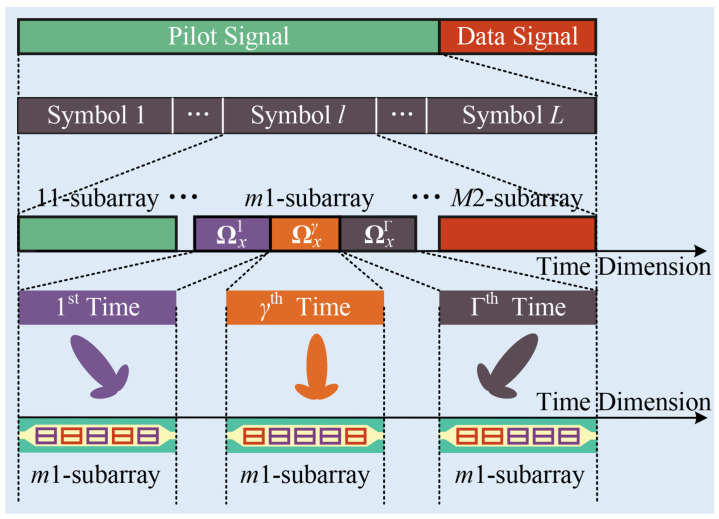
Time-division data reception scheme. The data frame is composed of *L* pilot symbols and the data signal. The *l*th symbol is observed by each subarray in sequence. For the m1-subarray, the weighted vectors vary across different times, generating distinct directional patterns that can be used to observe the symbol. The radiation pattern is altered Γ times to achieve multi-pattern observation. The total number of observing patterns is 2×M×Γ.

The Varactor diode-based DMA adjusts its capacitance by varying the biasing voltage, which subsequently alters the phase shift of each element. As shown in Figure 4, for the m1-subarray, the phase shift coefficients of each element vary Γ times within a single symbol duration. At the γth time, the element coefficients combine to form a unique pattern. The phase-shifted vector $\Omega_{x}^{\gamma }$ is expressed as $\Omega_{x}^{\gamma }={\left[e^{j\omega_{\gamma ,1}},\cdots ,e^{j\omega_{\gamma ,N-1}}\right]}^{T}$. The phase adjustment range is continuous, with $\omega_{\gamma ,n}=\left[-\pi ,\pi \right)$. Therefore, an appropriate weighting matrix $\Omega_{x}$ is needed to achieve finer control.

#### 3.1.2. Reception of Data Using Time-Division Scheme

To obtain the beamspace data through the proposed time-division scheme, we need to design the weighting matrix $\Omega_{x}$, where each vector enables the DMA to receive signals in a defined pattern.

DFT beamforming maximizes the number of resolvable path directions and eliminates ambiguity in the beamspace MUSIC spectrum [47]. Therefore, we utilize the DFT beamformer to design the phase shifts for each metamaterial element, resulting in distinct radiation patterns. Each subarray dynamically adjusts its radiation pattern based on the beamformer $\Omega_{x}$, defined by

(13) $$\Omega_{x}=\left[\nu \left(u_{1}\right),\cdots ,\nu \left(u_{\gamma }\right),\cdots ,\nu \left(u_{\Gamma }\right)\right],$$

where $u_{i}=u_{min}+\left(i-1\right)\times \left(u_{max}-u_{min}\right)/\left(\Gamma -1\right)$, i=1,2,⋯,Γ. Here, Γ represents the number of beams within the range $\left[u_{\min},u_{\max}\right]$ in the ROI [48]. Let the angle $u_{i}$ in *u*-space represent the *i*th direction. Then, $\nu (u_{i})$ is provided as follows:

(14) $$\nu \left(u_{i}\right)={\left[1,e^{j2\pi d_{x}u_{i}/\lambda },\cdots ,e^{j2\pi d_{x}u_{i}\left(N-2\right)/\lambda }\right]}^{T}.$$

**Figure 5:**
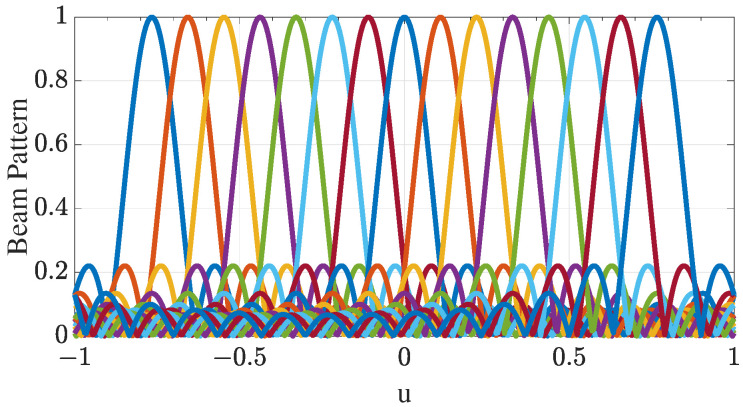
Beam patterns generated by the weighting matrix $\Omega_{x}$ within the ROI (ROI = $\left[-\sin\left(50^{\circ }\right),\sin\left(50^{\circ }\right)\right]$) in *u*-space. Given that N=16, the subarray has 15 elements, which can be used to form 15 beams, each represented by a different color and pointing to a different angle.

Similarly, $\Omega_{y}$ can be derived by treating the radiation pattern formed by $\nu (u_{i})$ as a ‘sinc’ function, where the peak response points in the direction of $u_{i}$, as shown in Figure 5. It is clear that the beam density in the range $\left[-\sin\left(50^{\circ }\right),\sin\left(50^{\circ }\right)\right]$ is larger than that in $\left[-\sin\left(90^{\circ }\right),\sin\left(90^{\circ }\right)\right]$. If the beam density in these two ranges were the same, the number of beams in $\left[-\sin\left(50^{\circ }\right),\sin\left(50^{\circ }\right)\right]$ could be smaller while achieving the same estimation performance. Therefore, with a narrow ROI, we can achieve better estimation performance with fewer beams by selecting the most appropriate number of beams.

Then, the received data $X^{B}$ of all subrows and received data $Y^{B}$ of all subcolumns are as shown below:

(15) $$X^{B}={\left[\Omega_{x}^{H}X_{11}^{e},\cdots ,\Omega_{x}^{H}X_{m1}^{e},\cdots ,\Omega_{x}^{H}X_{M2}^{e}\right]}^{T},$$

(16) $$Y^{B}={\left[\Omega_{y}^{H}Y_{11}^{e},\cdots ,\Omega_{y}^{H}Y_{n1}^{e},\cdots ,\Omega_{y}^{H}Y_{N2}^{e}\right]}^{T}.$$

### 3.2. Beamspace Spatial Smoothing Algorithm

The MUSIC algorithm cannot be directly applied in the case of coherent multipath signals, as it requires the data covariance matrix to be full-rank. Thus, a decorrelation procedure is necessary prior to implementing the MUSIC algorithm. In this section, we introduce the proposed BSS decorrelation technique, which is an improvement over Element-space Spatial Smoothing (ESS).

The covariance matrix $R_{m1}^{e}$ of the data $X_{m1}^{e}$ can be written as

(17) $$\begin{array}{cc} R_{m1}^{e} & =E[X_{m1}^{e}{X_{m1}^{e}}^{H}]\\ \; & =A_{m1}E\left[hss^{H}h^{H}\right]A_{m1}^{H}+\sigma^{2}I\\ \; & =A_{m1}R_{s}A_{m1}^{H}+\sigma^{2}I, \end{array}$$

where E[·] denotes the statistical expectation, $R_{s}$ represents the multipath signal covariance matrix, $\sigma^{2}$ represents the variance of the Gaussian white noise, and I denotes the (N−1)×(N−1) identity matrix.

The rank of the matrix $A_{m1}R_{s}A_{m1}^{H}$ for the m1-subarray is reduced to 1. The ESS technique uses data from 2M subarrays to enable rank restoration. The data covariance matrix $R_{x}^{ESS}$ then becomes

(18) $$R_{x}^{ESS}=\frac{1}{2M}\sum_{i=1}^{2}\sum_{m=1}^{M}R_{mi}^{e}.$$

However, the ESS technique based on traditional planar arrays is not applicable to the proposed DMA model. In contrast, the BSS technique is applicable to this model. BSS uses the received beamspace data to construct the following rank-restored data covariance matrix, $R_{x}^{BSS}\in C^{\Gamma \times \Gamma }$:

(19) $$\begin{array}{cc} R_{x}^{BSS} & =\frac{1}{2M}\sum_{i=1}^{2}\sum_{m=1}^{M}\Omega_{x}^{H}R_{mi}^{e}\Omega_{x}+\sigma^{2}\Omega_{x}^{H}\Omega_{x}\\ \; & =\frac{1}{2M}\sum_{i=1}^{2}\sum_{m=1}^{M}\Omega_{x}^{H}A_{m1}R_{s}A_{m1}^{H}\Omega_{x}+\sigma^{2}\Omega_{x}^{H}\Omega_{x}\\ \; & =\frac{1}{2M}\sum_{i=1}^{2}\sum_{m=1}^{M}\Omega_{x}^{H}A_{11}^{x}\Phi_{x}^{i-1}\Phi_{y}^{m-1}R_{s}{\left(\Phi_{y}^{m-1}\right)}^{H}{\left(\Phi_{x}^{i-1}\right)}^{H}{\left(A_{11}^{x}\right)}^{H}\Omega_{x}+\sigma^{2}\Omega_{x}^{H}\Omega_{x}\\ \; & =\Omega_{x}^{H}A_{11}^{x}\left[\frac{1}{2M}\sum_{i=1}^{2}\sum_{m=1}^{M}\Phi_{x}^{i-1}\Phi_{y}^{m-1}R_{s}{\left(\Phi_{y}^{m-1}\right)}^{H}{\left(\Phi_{x}^{i-1}\right)}^{H}\right]{\left(A_{11}^{x}\right)}^{H}\Omega_{x}+\sigma^{2}\Omega_{x}^{H}\Omega_{x}\\ \; & =\Omega_{x}^{H}A_{11}^{x}{\bar{R}}_{s}{\left(A_{11}^{x}\right)}^{H}\Omega_{x}+\sigma^{2}\Omega_{x}^{H}\Omega_{x} \end{array}$$

where ${\bar{R}}_{s}$ is the modified covariance matrix of the signals, provided by

(20) $${\bar{R}}_{s}=\frac{1}{2M}\sum_{i=1}^{2}\sum_{m=1}^{M}\Phi_{x}^{i-1}\Phi_{y}^{m-1}R_{s}{\left(\Phi_{y}^{m-1}\right)}^{H}{\left(\Phi_{x}^{i-1}\right)}^{H}.$$

**Lemma** **1.** *If the number of subarrays is greater than or equal to the number of multipath signals, i.e., if 2M≥K, then the modified covariance matrix of the signals ${\bar{R}}_{s}$ is nonsingular.*

**Proof.**  First, note that we can rewrite ${\bar{R}}_{s}$ as

(21) $${\bar{R}}_{s}=\left[I,\Phi_{y},\cdots ,\Phi_{y}^{M-1},\Phi_{x},\cdots ,\Phi_{x}\Phi_{y}^{M-1}\right]\left[\begin{array}{ccc} \frac{1}{2M}R_{s} & \; & \\ \; & \ddots & \\ \; & \; & \frac{1}{2M}R_{s} \end{array}\right]\left[\begin{array}{c} I\\ \Phi_{y}^{H}\\ \vdots \\ {\left(\Phi_{y}^{M-1}\right)}^{H}\\ {\left(\Phi_{x}\right)}^{H}\\ \vdots \\ {\left(\Phi_{x}\Phi_{y}^{M-1}\right)}^{H} \end{array}\right],$$

which can be further simplified to

(22) $${\bar{R}}_{s}=GG^{H},$$

where G is the K×2MK block matrix

(23) $$G=[C,\Phi_{y}C,\cdots ,\Phi_{y}^{M-1}C,\Phi_{x}C,\Phi_{x}\Phi_{y}C,\cdots ,\Phi_{x}\Phi_{y}^{M-1}C],$$

in which C denotes the Hermitian square root of $\frac{1}{2M}R_{s}$: $CC^{H}=\frac{1}{2M}R_{s}$. Clearly, the rank of ${\bar{R}}_{s}$ is equal to the rank of G. If 2M≥K, the rank of modified covariance matrix ${\bar{R}}_{s}$ is *K*, and ${\bar{R}}_{s}$ is nonsingular. □

Therefore, it can be seen that the rank of the beamspace data covariance matrix $R_{x}^{BSS}$ is equal to *K*. Similarly, we can derive $R_{y}^{BSS}\in C^{\Gamma \times \Gamma }$. Compared with ESS, the BSS technique improves the DOF and yields better angular resolution for coherent signals. Then, the beamspace MUSIC algorithm can be employed to estimate the DOA.

### 3.3. Beamspace MUSIC Algorithm

With the rank-restored data covariance matrix, the beamspace MUSIC algorithm can be applied for DOA estimation. The subspace decomposition of $R_{x}^{BSS}$ is

(24) $$R_{x}^{BSS}=\sum_{\gamma =1}^{\Gamma }\lambda_{x_{\gamma }}e_{x_{\gamma }}e_{x_{\gamma }}^{H}=E_{s_{x}}\Lambda_{s_{x}}E_{s_{x}}^{H}+E_{w_{x}}\Lambda_{w_{x}}E_{w_{x}}^{H},$$

where $\lambda_{x_{1}}\geq \lambda_{x_{2}}\geq \cdots \geq \lambda_{x_{K}}\geq \lambda_{x_{K+1}}\geq \cdots \geq \lambda_{x_{\Gamma }}$ are the eigenvalues of $R_{x}^{BSS}$ and $e_{x_{\gamma }}$ is the $\gamma^{th}\left(\gamma =1,2,\cdots ,\Gamma \right)$ corresponding orthonormal eigenvector. In addition, $E_{s_{x}}=\left[e_{x_{1}},e_{x_{2}},\cdots ,e_{x_{K}}\right]$ with rank *K* spans the signal subspace and $E_{w_{x}}=\left[e_{x_{K+1}},e_{x_{K+2}},\cdots ,e_{x_{\Gamma }}\right]$ spans the noise subspace, while $\Lambda_{s_{x}}$ and $\Lambda_{w_{x}}$ are diagonal matrices with the respective eigenvalues along the main diagonal. Carrying out the same EVD decomposition on $R_{y}^{BSS}$, the beamspace MUSIC spatial spectra for the *x*-axis and *y*-axis directions are provided by Equations (25) and (26), respectively. Then, the corresponding angles $\hat{\alpha }$ and $\hat{\beta }$ are found as follows:

(25) $$P_{x-MUSIC}\left(\alpha \right)=\frac{a_{x}^{H}\left(\alpha \right)\Omega_{x}\Omega_{x}^{H}a_{x}\left(\alpha \right)}{a_{x}^{H}\left(\alpha \right)\Omega_{x}E_{n_{x}}E_{n_{x}}^{H}\Omega_{x}^{H}a_{x}\left(\alpha \right)}$$

(26) $$P_{y-MUSIC}\left(\beta \right)=\frac{a_{y}^{H}\left(\beta \right)\Omega_{y}\Omega_{y}^{H}a_{y}\left(\beta \right)}{a_{y}^{H}\left(\beta \right)\Omega_{y}E_{n_{y}}E_{n_{y}}^{H}\Omega_{y}^{H}a_{y}\left(\beta \right)}$$

where $a_{x}\left(\alpha \right)={\left[1,\cdots ,e^{j2\pi d_{x}\cos\alpha \left(N-2\right)/\lambda }\right]}^{T}$ and $a_{y}\left(\beta \right)={\left[1,\cdots ,e^{j2\pi d_{y}\cos\beta \left(N-2\right)/\lambda }\right]}^{T}$.

The angles $\hat{\theta }$ and $\hat{\phi }$ of the multipath signals are obtained by performing calculations based on the geometric relationship, as shown in Equation (27):

(27) $$\left\{\begin{array}{cc} {\hat{\theta }}_{k} & =\arcsin\sqrt{\cos^{2}{\hat{\alpha }}_{k}+\cos^{2}{\hat{\beta }}_{k}}\\ {\hat{\phi }}_{k} & =\arctan\frac{\cos{\hat{\beta }}_{k}}{\cos{\hat{\alpha }}_{k}}. \end{array}\right.$$

Because the estimated angles $\hat{\theta }$ and $\hat{\phi }$ are obtained independently, we reconstruct the element-space array data to pair the multipath DOAs using the conventional 2D SS-MUSIC algorithm [16]. The 2D SS-MUSIC algorithm divides the array into overlapping subarrays with $\bar{M}$ elements per row and $\bar{N}$ elements per column. Then, the spatial smoothed covariance matrix $\bar{R}$ is used to estimate the DOAs.

We define the $\left(\bar{m},\bar{n}\right)$ 2D subarray data $Z\in C^{F\times L}$ as shown below:

(28) $$\begin{array}{c} Z=\bar{A}hs+\bar{W}=\left[\begin{array}{c} {\bar{A}}_{x}\\ {\bar{A}}_{x}\Phi_{y}\\ \vdots \\ {\bar{A}}_{x}\Phi_{y}^{\bar{M}-1} \end{array}\right]\Phi_{x}^{\bar{n}-1}\Phi_{y}^{\bar{m}-1}hs+\bar{W} \end{array}$$

where $\bar{m}\leq M-\bar{M}+1$, $\bar{n}\leq N-\bar{N}+1$, $F=\bar{M}\times \bar{N}$, ${\bar{A}}_{x}={\left[1,\cdots ,e^{j2\pi d_{x}u_{i}\left(\bar{N}-1\right)/\lambda }\right]}^{T}$.

The size of the 2D subarray is $\bar{M}\times \bar{N}$, meaning that the total number of 2D subarrays is provided by $B=\left(M-\bar{M}+1\right)\times \left(N-\bar{N}+1\right)$. Here, we define $P=\bar{M}\times \bar{N}$. The covariance matrix of the *b*th subarray is ${\bar{R}}_{b}$. The spatial smoothed covariance matrix is expressed as

(29) $$\bar{R}=\frac{1}{B}\sum_{b=1}^{B}{\bar{R}}_{b}=\sum_{p=1}^{P}{\bar{\lambda }}_{p}{\bar{e}}_{p}{\bar{e}}_{p}^{H}={\bar{E}}_{s}{\bar{\Lambda }}_{s}{\bar{E}}_{s}^{H}+{\bar{E}}_{w}{\bar{\Lambda }}_{w}{\bar{E}}_{w}^{H},$$

where ${\bar{\lambda }}_{1}\geq {\bar{\lambda }}_{2}\geq \cdots \geq {\bar{\lambda }}_{K}\geq {\bar{\lambda }}_{K+1}\geq \cdots \geq {\bar{\lambda }}_{P}$ are the eigenvalues of $\bar{R}$ and ${\bar{e}}_{i}$ is the $p^{th}\left(p=1,2,\cdots ,P\right)$ corresponding orthonormal eigenvector. In addition, ${\bar{E}}_{s}=\left[{\bar{e}}_{1},{\bar{e}}_{2},\cdots ,{\bar{e}}_{K}\right]$ with rank *K* spans the signal subspace and ${\bar{E}}_{w}=\left[{\bar{e}}_{K+1},{\bar{e}}_{K+2},\cdots ,{\bar{e}}_{P}\right]$ spans the noise subspace, while ${\bar{\Lambda }}_{s}$ and ${\bar{\Lambda }}_{w}$ are diagonal matrices with the respective eigenvalues along the main diagonal.

Eigenvalue decomposition of $\bar{R}$ is conducted in order to ascertain the signal and noise subspaces. Subsequently, the elevation and azimuth can be determined through a 2D MUSIC search, as outlined below:

(30) $$P_{2D-MUSIC}\left(\theta ,\phi \right)=\frac{{\bar{a}}^{H}\left(\theta ,\phi \right)\bar{a}\left(\theta ,\phi \right)}{{\bar{a}}^{H}\left(\theta ,\phi \right){\bar{E}}_{w}{\bar{E}}_{w}^{H}\bar{a}\left(\theta ,\phi \right)}$$

where $\bar{a}\left(\theta ,\phi \right)=\left[{\bar{a}}_{y}\left(\theta ,\phi \right)⊗{\bar{a}}_{x}\left(\theta ,\phi \right)\right]$, ${\bar{a}}_{x}\left(\theta ,\phi \right)={\left[1,e^{j2\pi d_{x}\cos\alpha /\lambda },\cdots ,e^{j2\pi d_{x}\cos\alpha \left(\bar{N}-1\right)/\lambda }\right]}^{T}$, ${\bar{a}}_{y}\left(\theta ,\phi \right)={\left[1,e^{j2\pi d_{y}\cos\beta /\lambda },\cdots ,e^{j2\pi d_{y}\cos\beta \left(\bar{M}-1\right)/\lambda }\right]}^{T}$.

As there are K×K pairs for $\hat{\theta }$ and $\hat{\phi }$, we choose the *K* pairs corresponding to the *K* largest values by finding the peaks through Equation (30).

In this study, without loss of generality, we employ the least squares fitting algorithm. The channel estimation result can be calculated as

(31) $$\hat{h}\left(\hat{\theta },\hat{\phi }\right)=\frac{{\bar{A}}^{†}Zs^{H}}{{∥s∥}_{2}}.$$

### 3.4. Complexity Analysis

Next, we provide a detailed computational complexity analysis. The complexity is evaluated by computing only the complex multiplications, which mainly include two parts, namely, the estimation procedure and angle pairing procedure.

In the estimation procedure, we primarily analyze the computational complexity of three fundamental operations: construction of the full rank covariance matrix, EVD, and spectral peak searching. The complexity is $\max\left\{O\left[\left(2K^{2}+2M\right)\Gamma^{2}\right],O\left[\left(2K^{2}+2N\right)\Gamma^{2}\right]\right\}+O\left(\Gamma^{3}\right)+O\left(G\Gamma^{3}\right)$, where *G* represents the total number of angle grid points used for searching. The complexity of the angle pairing procedure is $O(K^{2}{\bar{M}}^{2}{\bar{N}}^{2}+{\bar{M}}^{3}{\bar{N}}^{3})$.

The number of grid points for spectral peak searching using the 2D-MUSIC algorithm is $G^{2}$, while the number of grid points when using the BSS-MUSIC or ESS-MUSIC algorithms is 2G, resulting in a significant reduction in the number of search points. Moreover, as the number of beams is usually smaller than the number of elements (i.e., K<Γ<min{M,N}), the computational complexity of BSS-MUSIC is lower than that of ESS-MUSIC.

## 4. Simulation Results

In this section, we demonstrate the performance of the proposed method to show its rationality and effectiveness. We compare our proposed BSS-MUSIC method with the OMP method as well as with MUSIC methods based on existing spatial smoothing algorithms such as FSS [10], FBSS [11], and ESS [43]. Our simulation results show that dynamic selection of the observation area can significantly improve the performance of DOA estimation.

(32) $$\mathrm{NMSE}\left(\theta \right)=\frac{E\left[{∥\theta -\hat{\theta }∥}_{2}^{2}\right]}{E\left[{∥\theta ∥}_{2}^{2}\right]}.$$

(33) $$\mathrm{NMSE}\left(\phi \right)=\frac{E\left[{∥\phi -\hat{\phi }∥}_{2}^{2}\right]}{E\left[{∥\phi ∥}_{2}^{2}\right]}.$$

(34) $$\mathrm{NMSE}\left(h\left(\theta ,\phi \right)\right)=\frac{E\left[{∥h\left(\theta ,\phi \right)-\hat{h}\left(\theta ,\phi \right)∥}_{2}^{2}\right]}{E\left[{∥h\left(\theta ,\phi \right)∥}_{2}^{2}\right]}.$$

The deviation between the estimated and actual values is evaluated using the Normalized Mean Squared Error (NMSE), which is influenced by critical parameters such as the Signal-to-Noise Ratio (SNR), array resolution, and errors in the array element positions. Additionally, we validated the impact of the number of beams on estimation performance. We report the NMSE values for both the DOA and complex gain.

To validate the proposed scheme, we conducted Monte Carlo simulations. Unless stated otherwise, the simulation parameters were those summarized in Table 1.

### 4.1. Impact of SNR

The SNR is one of the critical parameters influencing DOA estimation performance. In this section, we examine the performance of DOA estimation following SNR enhancement or reduction. There are two ways to improve SNR. The first involves increasing the transmit power or reducing the noise power by increasing the number of pilot symbols. The SNR of each multipath signal may decrease when the transmit power allocated to each path is reduced as the total number of paths increases.

#### 4.1.1. Effect of Increasing Transmit Power

**Figure 6:**
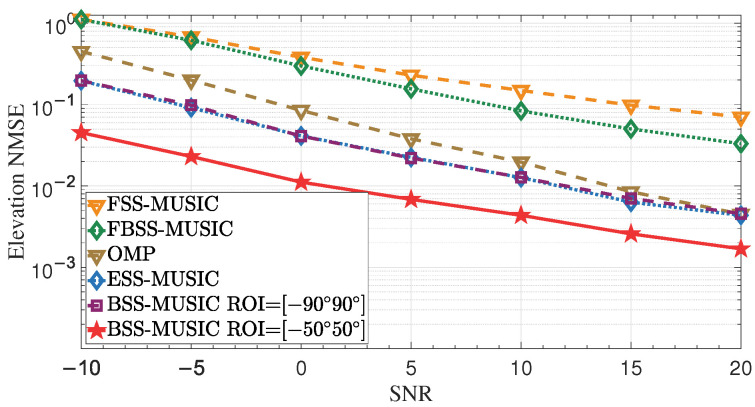

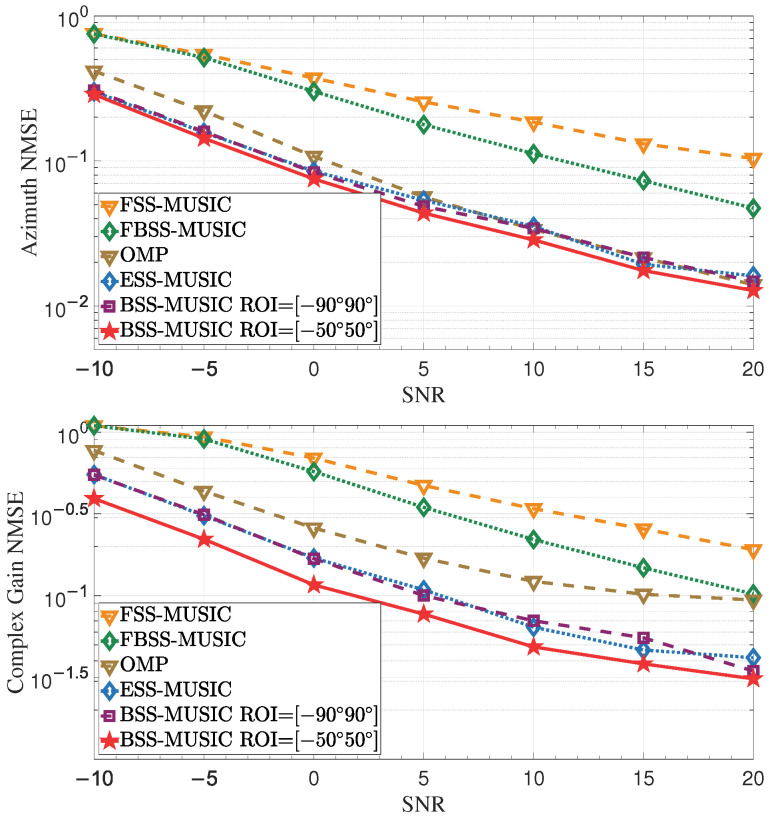
NMSE of DOA and complex gain over the SNR. Higher SNR results in smaller NMSE. It can be seen that BSS-MUSIC demonstrates superior performance when the ROI ranges from $-50^{\circ }$ to $50^{\circ }$.

This simulation was used to assess the 2D DOA estimation error at various transmit powers. The transmit power range was from −10 to 20 dBm with a 5 dB step, and the noise power was 0 dBm; thus, the SNR range was from −10 to 20 dB. As shown in Figure 6, the NMSE gradually decreased with increasing SNR. The solid line with star markers shows that the performance of BSS-MUSIC with ROI $\left[-50^{\circ },50^{\circ }\right]$ is the best. In this case, concentration of the angle-of-observation patterns within the ROI range helps to improve the resolution. BSS-MUSIC with ROI $\left[-90^{\circ },90^{\circ }\right]$ and ESS-MUSIC both have the same estimation performance when the arrays have the same array aperture, as demonstrated by the two curves in the central region. However, it should be noted that the cost of the DMA is significantly reduced because it only has one RF chain, while the 32×32 URA has 1024 RF chains. In multi-snapshot scenarios, the performance of OMP is inferior to that of MUSIC. The performance of FBSS-MUSIC and FSS-MUSIC is also worse than that of other methods, as traditional spatial smoothing algorithms lead to significant loss of array aperture.

#### 4.1.2. Effect of Increasing Pilot Length

**Figure 7:**
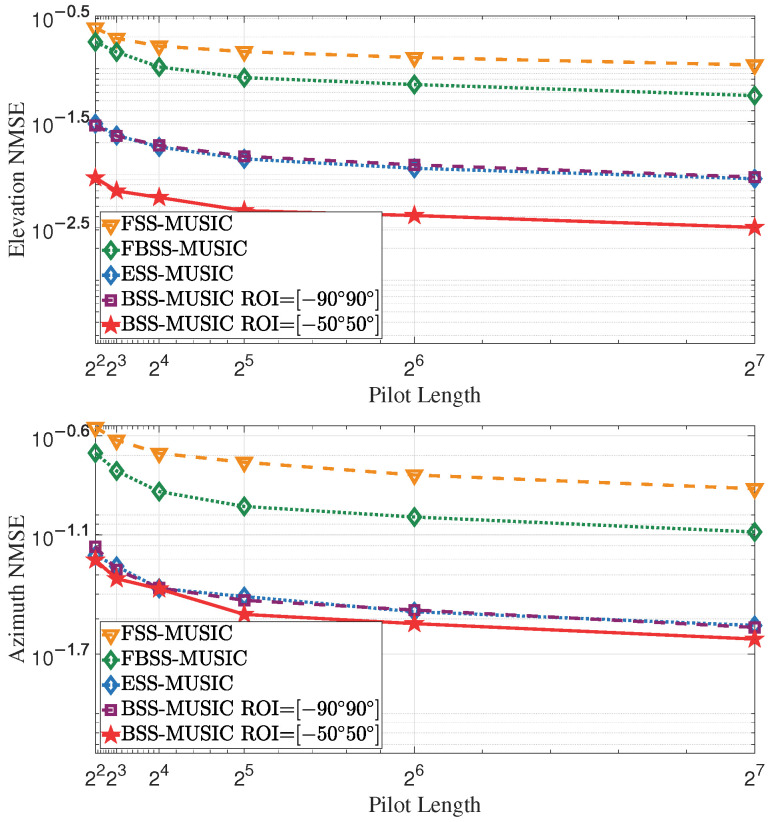

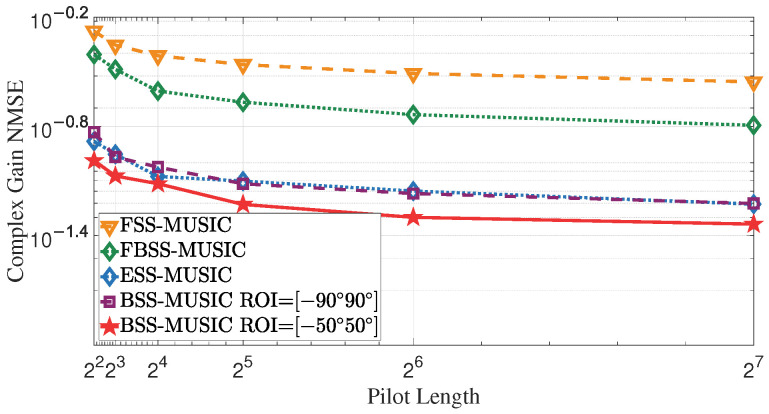
NMSE of DOA and complex gain over the pilot length. A longer pilot length helps to reduce the NMSE, but also introduces higher overhead, which consequently lowers the communication rate. Therefore, it is important to choose an appropriate number of pilots.

This simulation was used to assess the estimation error at various pilot lengths ranging from $2^{2}$ to $2^{7}$. The SNR was 10 dB. Increasing the pilot length is equivalent to increasing the number of samples for averaging; therefore, as shown in Figure 7, a longer pilot sequence provides better noise reduction, leading to improved estimation accuracy. However, the NMSE does not decrease indefinitely as the number of pilots increases. Moreover, longer pilot lengths introduce higher overhead. Therefore, it is important to choose an appropriate number of pilots that balances computational effort and estimation accuracy.

#### 4.1.3. Effect of Increasing Multipaths Number

**Figure 8:**
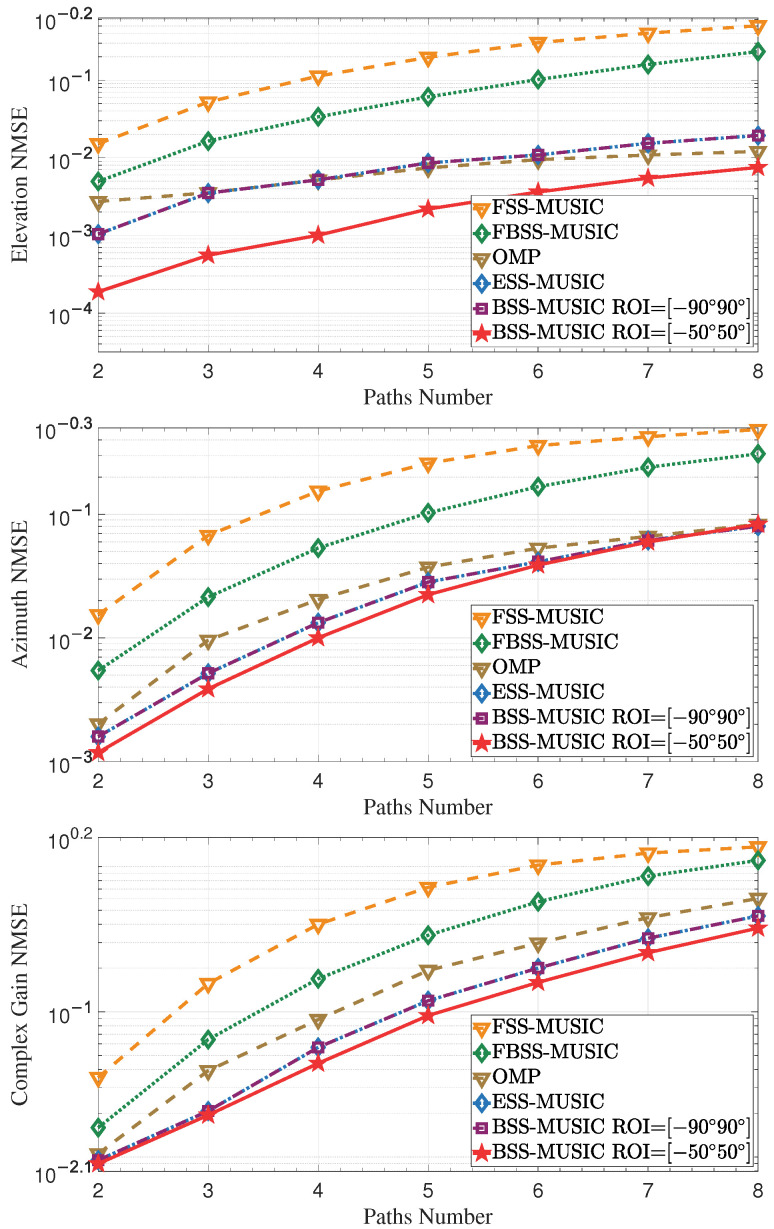
NMSE of DOA and complex gain over the multipaths number. As the number of multipaths increases, the power allocated to each is reduced, leading to an increase in NMSE.

This simulation was used to assess the estimation performance with various multipaths numbers. The simulation parameters of this experiment consisted of 64 microstrips and 64 array elements for each microstrip. The multipaths number was changed from 2 to 8 with a step of 1, with all other parameters staying the same. Figure 8 illustrates that the NMSE increases as the number of multipaths increases with the total power fixed, leading to a decrease in the power allocated to each multipath.

### 4.2. Impact of the Array Resolution

The resolution is another critical parameter influencing estimation performance. It refers to the ability of the DMA to distinguish the multipath signals arriving from different angles even when those angles are very similar. The number of elements and incident angle range both affect the array resolution.

#### 4.2.1. Effect of Increasing Number of Elements

**Figure 9:**
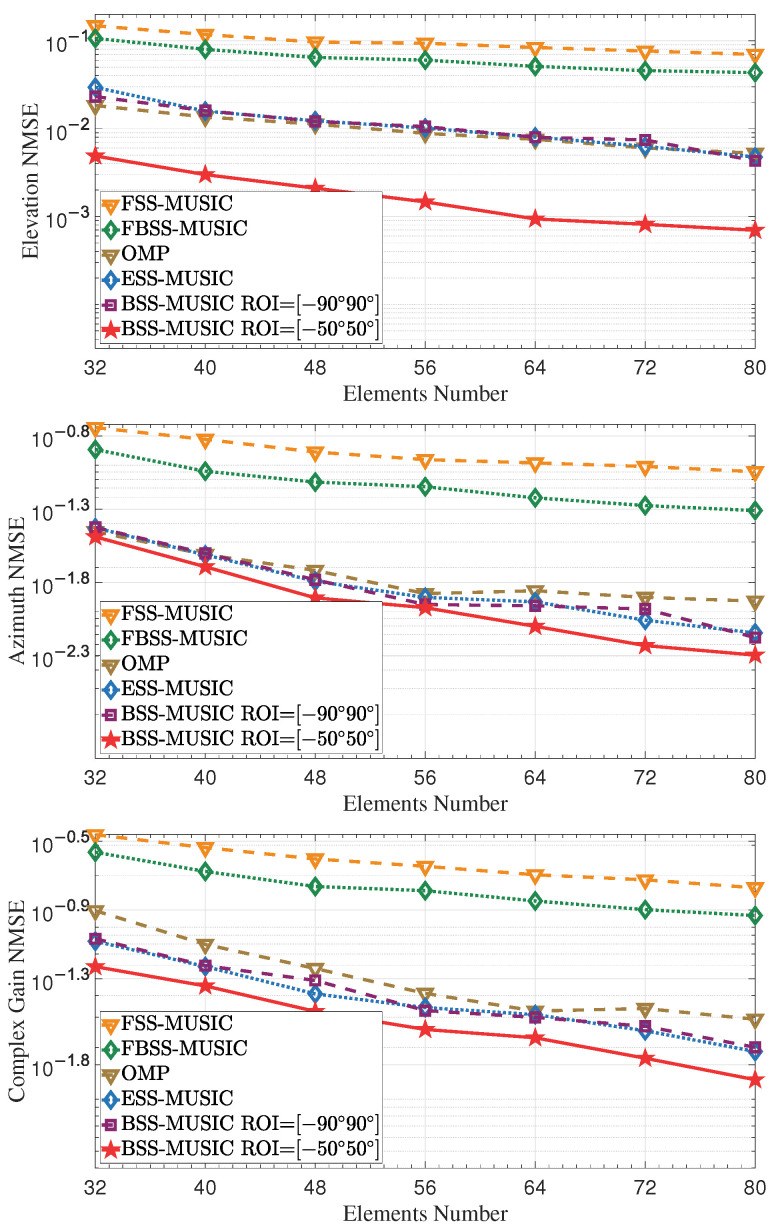
NMSE of DOA and complex gain over the elements number. As the number of microstrips and elements increases, the NMSE gradually decreases.

This simulation was used to assess the estimation error with various element numbers, as shown in Figure 9. More microstrips and elements lead to better performance. As the number of elements increases, the main lobe width of the array pattern becomes narrower, yielding the benefits of higher resolution and higher estimated accuracy. However, URAs need many more RF chains as the number of elements increases, making the array too expensive to have practical value. Fortunately, DMAs add metamaterial elements and fewer RF chains, meaning that the cost does not increase very much. Moreover, it is evident that the performance of BSS-MUSIC with the ROI set to $\left[-50^{\circ },50^{\circ }\right]$ is the best, as dynamic selection of the ROI helps to improve estimation performance.

#### 4.2.2. Effect of Incident Angle Range

**Figure 10:**
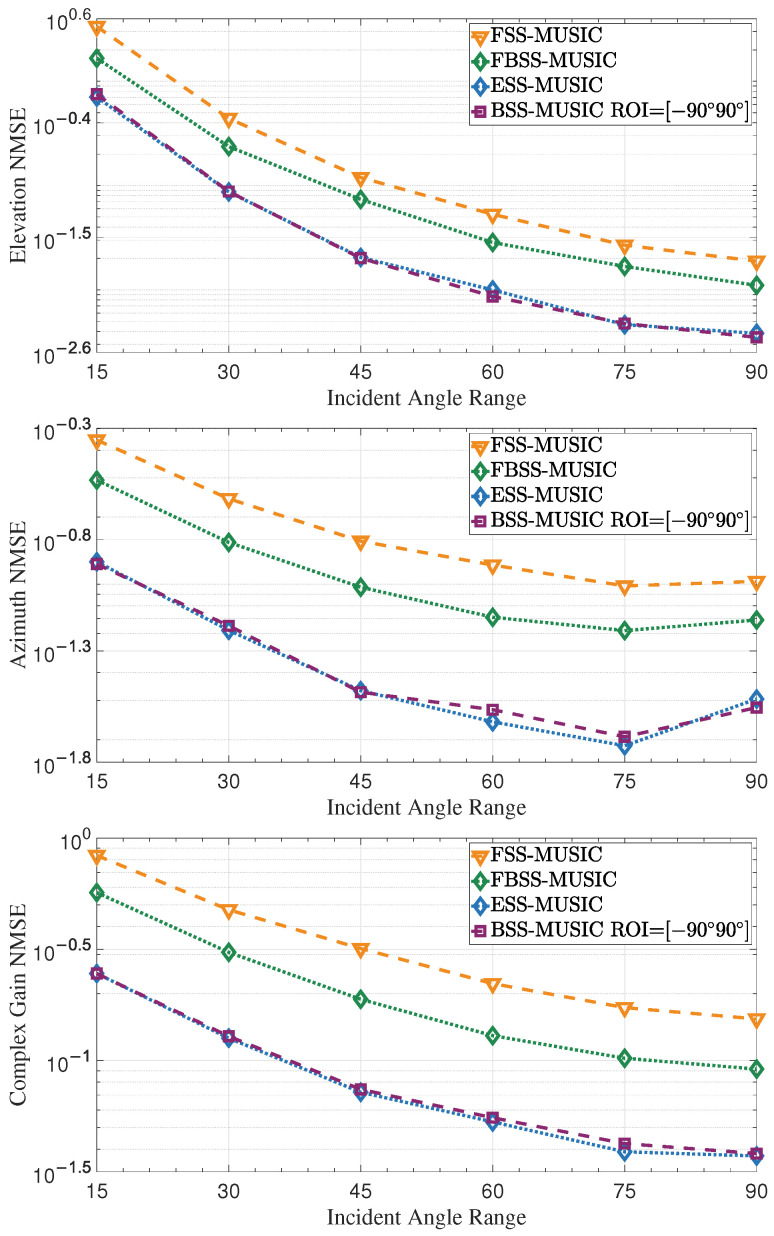
NMSE of DOA and complex gain over the incident angle range. The angle interval of the multipath signals and the array resolution both affect the estimation accuracy. When the range is small, the angle interval is narrow, resulting in a higher NMSE. When the range is large, the resolution decreases as the angle increases, leading to a slightly higher NMSE.

As shown in Figure 10, the NMSE exhibits a gradual change as the incident angle range increases. When the range is small, such as $\left[-15^{\circ },15^{\circ }\right]$, the limited range leads to a high NMSE because the array has difficulty distinguishing between two closely spaced multipaths. As the range increases, for example to $\left[-45^{\circ },45^{\circ }\right]$, the larger angle interval makes it easier for the array to distinguish DOAs, resulting in improving performance. However, when the range extends to $\left[-90^{\circ },90^{\circ }\right]$, some multipath angles may exceed $60^{\circ }$, as the array resolution decreases with increasing angle, which leads to a high NMSE. Therefore, the curve exhibits an upward trend beyond $70^{\circ }$. If there is only one path, smaller the θ makes for better DOA estimation accuracy.

### 4.3. Impact of Array Element Position Error

**Figure 11:**
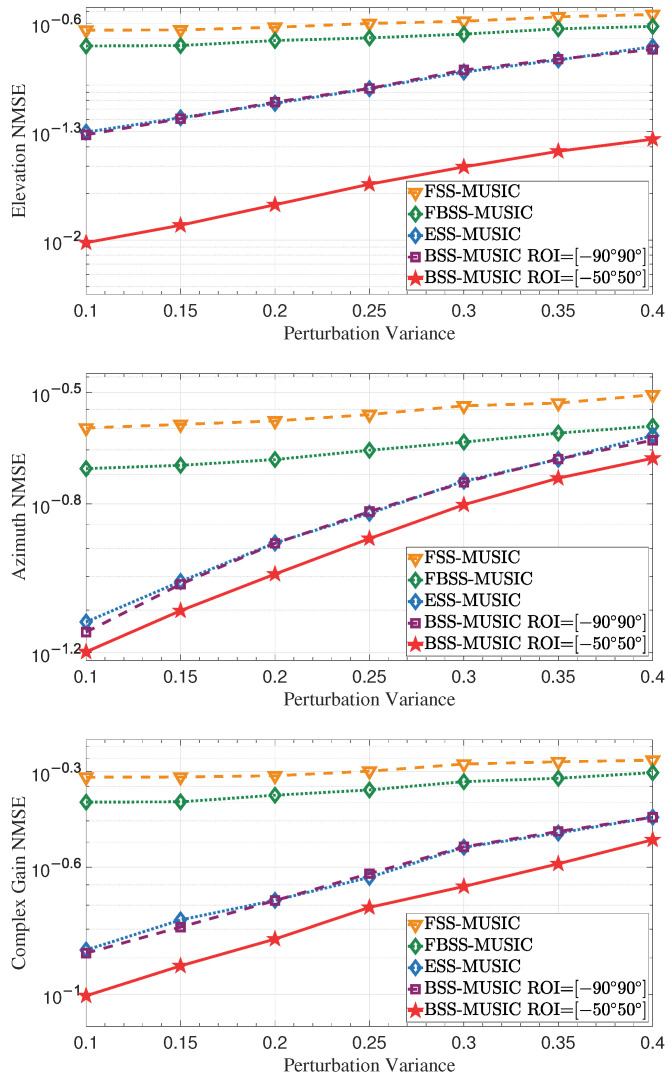
NMSE of the DOA and complex gain with different error variance. Deviation in the positions of array elements results in an increase in NMSE.

Position error also has a significant impact on estimation performance, reducing the accuracy of the algorithms. In this simulation, we set the position error variance to range from 0.1 to 0.4 [49], with a step size of 0.05. As shown in Figure 11, the estimation performance gradually deteriorates as the error variance increases. Accurate knowledge of the array manifold is used to implement the MUSIC algorithm. However, position error introduces phase jitter into the array manifold vectors, degrading the estimation performance. Compared with the conventional MUSIC algorithms, BSS-MUSIC is not as sensitive to position error, and consequently offers better estimation performance.

### 4.4. Effect of Beam Number

**Figure 12:**
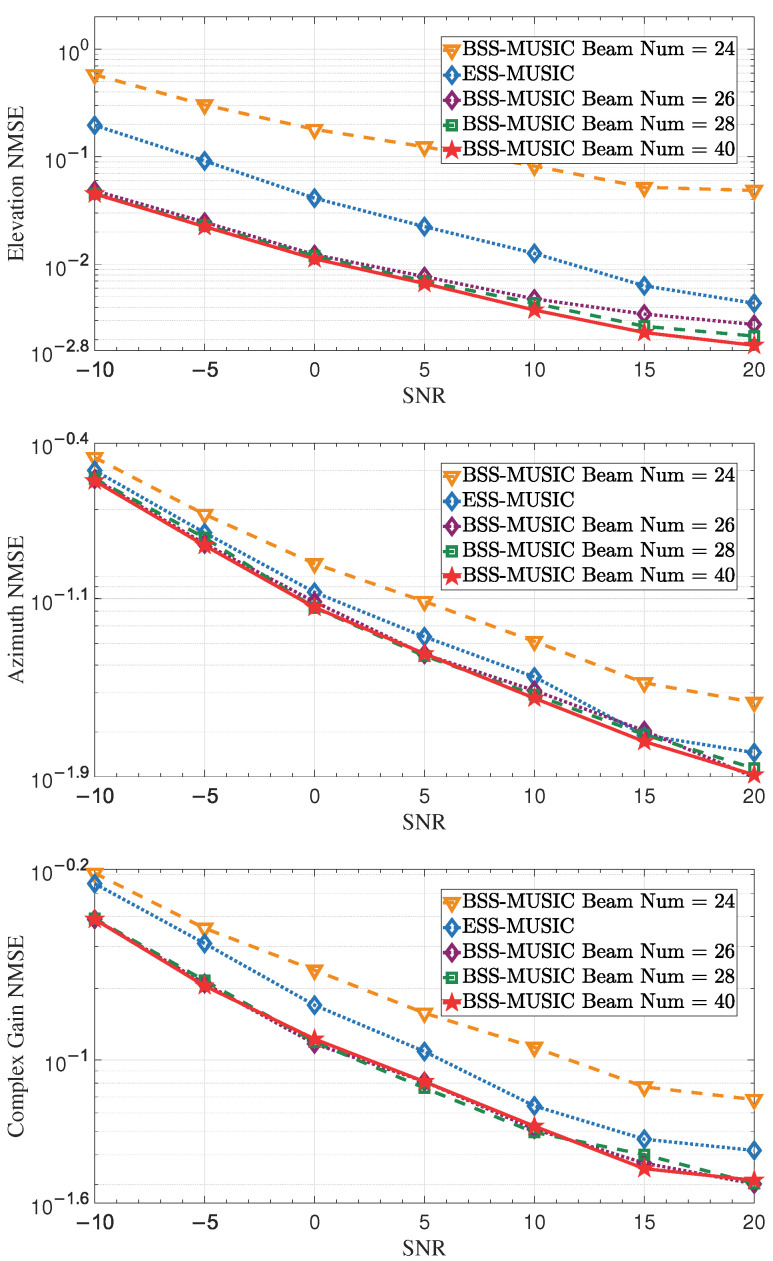
NMSE of DOA and complex gain over the SNR with different beam numbers. For a given ROI, performance improves with an increasing number of beams; however, the performance gains eventually plateau as the number of beams increases due to the constraints imposed by the array aperture.

This simulation aimed to evaluate the estimation error at various SNRs using different numbers of beams. We verified the performance using beams numbers of 24, 26, and 28, all of which are smaller than the number of subarray elements. Additionally, we selected a beam number of 40, which exceeds the number of subarray elements [50]. The ROI range was from $-50^{\circ }$ to $50^{\circ }$. As shown in Figure 12, the more beams there are, the better the performance; however, it can also be observed that the curves for the numbers 26, 28, and 40 move forward alternately. The proximity of these curves indicates that performance does not always improve as the number of beams increases, which is due to the limitations of the array aperture. Meanwhile, selecting a beam number such as 26 or 28 helps to reduce computational complexity without degrading performance.

## 5. Conclusions

In this paper, we propose a BSS-MUSIC DOA estimation method using a DMA. We exploit the advantages of the on/off nature and pattern agility of DMAs to propose a time-division data reception scheme designed to capture beamspace data for DOA estimation. Then, the BSS algorithm is proposed to decohere the multipath signals and restore the rank of the beamspace data covariance matrix. Next, the 1D beamspace MUSIC algorithm is used to estimate the DOA against the *x*-axis and *y*-axis. After pairing the elevation and azimuth angles, the complex gain of each path is derived. Simulation results demonstrate that the proposed method can effectively achieve superior DOA and complex gain estimation compared to conventional methods. Notably, this method only requires a single RF chain, in contrast to traditional methods that require M×N RF chains. Furthermore, the proposed time-division data reception scheme can effectively enhance spatial and temporal DOF, enabling dynamic selection of the ROI range and dynamic adjustment of the beam number. Within the selected ROI, this method reduces computational complexity without sacrificing estimation accuracy.

## Figures and Tables

**Table 1:** Simulation parameters.

Parameter Type	Parameter Value
Number of Microstrips *M*	32
Number of Elements *N*	32
Element Spacing $d_{x}=d_{y}$	λ/2
Number of Desired Signal	3
Number of Snapshots	32
Elevation Range θ	$\left[5^{\circ },45^{\circ }\right]$
Azimuth Range ϕ	$\left[-120^{\circ },120^{\circ }\right]$
Monte-Carlo	10,000

## Data Availability

The original contributions presented in this study are included in the article. Further inquiries can be directed to the corresponding author.

## Abbreviations

The following abbreviations are used in this manuscript:

2D	Two-dimensional
BSS-MUSIC	Beamspace spatial smoothing multiple signal classification
CS	Compressed sensing
DMA	Dynamic metasurface antenna
DOA	Direction-of-arrival
DOF	Degrees of freedom
ESS	Element-space spatial smoothing
ESPRIT	Estimation of signal parameters via rotational invariance techniques
FBSS	Forward/backward spatial smoothing
FSS	Forward spatial smoothing
IQML	Iterative quadratic maximum likelihood
ML	Maximum likelihood
MODE	Method of direction estimation
NMSE	Normalized mean squared error
OMP	Orthogonal matching pursuit
ROI	Region of interest
SNR	Signal-to-noise ratio
SS	Spatial smoothing
URA	Uniform rectangular arrays
WSF	Weighted subspace fitting
